# Meeting Report: Controlled Human Influenza Virus Infection Model Studies: Current Status and Future Directions for Innovation

**DOI:** 10.1111/irv.13358

**Published:** 2024-10-23

**Authors:** M. Chelsea Lane, Catherine J. Luke, Joseph Bresee, Vivien G. Dugan, Diane J. Post, Julie Schafer, Paul C. Roberts, David E. Wentworth, Michael G. Ison

**Affiliations:** ^1^ Division of Microbiology and Infectious Diseases National Institute of Allergy and Infectious Diseases Bethesda Maryland USA; ^2^ The Task Force for Global Health Decatur Georgia USA; ^3^ Centers for Disease Control and Prevention Atlanta Georgia USA; ^4^ Flu Lab Palo Alto California USA

**Keywords:** controlled human infection model (CHIM), controlled human infection model (CHIVIM), influenza vaccines, influenza virus, influenza virus

## Abstract

On November 13–14, 2023, the National Institute of Allergy and Infectious Diseases (NIAID) in partnership with the Task Force for Global Health, Flu Lab, the Canadian Institutes of Health Research, and the Centers for Disease Control and Prevention convened a meeting on controlled human influenza virus infection model (CHIVIM) studies to review the current research landscape of CHIVIM studies and to generate actionable next steps. Presentations and panel discussions highlighted CHIVIM use cases, regulatory and ethical considerations, innovations, networks and standardization, and the utility of using CHIVIM in vaccine development. This report summarizes the presentations, discussions, key takeaways, and future directions for innovations in CHIVIMs. Experts agreed that CHIVIM studies can be valuable for the study of influenza infection, immune response, and transmission. Furthermore, they may have utility in the development of vaccines and other medical countermeasures; however, the use of CHIVIMs to de‐risk clinical development of investigational vaccines should employ a cautious approach. Endpoints in CHIVIM studies should be tailored to the specific use case. CHIVIM studies can provide useful supporting data for vaccine licensure but are not required and do not obviate the need for the conduct of field efficacy trials. Future directions in this field include the continued expansion of capacity to conduct CHIVIM studies, development of a broad panel of challenge viruses and assay reagents and standards that can be shared, streamlining of manufacturing processes, the exploration of targeted delivery of virus to the lower respiratory tract, efforts to more closely replicate natural influenza disease in CHIVIM, alignment on a definition of breadth to facilitate development of more broadly protective/universal vaccine approaches, and continued collaboration between stakeholders.

## 1 Introduction

In 2018, the Bill & Melinda Gates Foundation (BMGF), PATH, and the Global Funders Consortium for Universal Influenza Vaccine Development convened a meeting in London, UK, to collect perspectives and identify the best practices on how to improve the utility of a standardized controlled human influenza virus infection model (CHIVIM) [1, 2]. Steps were identified to develop CHIVIM for universal influenza vaccine research with the ultimate goal of acceleration of the accumulation of knowledge needed to develop and license broadly protective influenza vaccines offering long‐lasting protection against both seasonal and pandemic disease. These steps include (1) standardization of the model, (2) manufacturing of challenge virus stocks, (3) risk mitigation, (4) development of a research network and refinement of the objective measures of influenza disease, (5) expansion of access and capacity for CHIVIM studies, and (6) further refinement of CHIVIM as results become available.

In follow‐up to the 2018 CHIVIM meeting, the National Institute of Allergy and Infectious Diseases (NIAID) in partnership with the Task Force for Global Health, Flu Lab, the Canadian Institutes of Health Research, and the Centers for Disease Control and Prevention convened a meeting on CHIVIM studies to review the current research landscape and to generate actionable next steps for model development and use. Presentations and panel discussions highlighted CHIVIM use cases, regulatory and ethical considerations, innovations, networks and standardization, and utility of using CHIVIM in vaccine development. In moving forward, the broader influenza community should continue to (1) evaluate the need for CHIVIM in evaluating innovative next‐generation influenza vaccine candidates; (2) navigate ethical and regulatory landscapes pertaining to CHIVIM; (3) refine and innovate CHIVIM studies; (4) standardize protocols, reagents, and immunological assays as needed; and (5) foster local, national, and global collaborations. See Appendix for the full meeting agenda.

## 2 Session 1: CHIVIM: Progress, Updates, and Lessons Since the 2018 Meeting

Since the 2018 CHIVIM meeting, significant progress has been made in the universal influenza vaccine and controlled human infection model (CHIM) fields. There have been extensive discussions on target product profiles for universal influenza vaccines, and many candidate vaccines are in clinical development, several of which are moving into early phase clinical trials [3]. CHIVIM studies can help overcome hurdles that persist in influenza vaccine development: limited utility of animal models, need to identify clear correlates of protection, and may aid in de‐risking/down‐selection during clinical development. The strengths of CHIVIM include the ability to perform rapid, flexible controlled studies that can provide early reads on effectiveness of countermeasures and the ability to define static and dynamic host protective factors through longitudinal analysis.

Several consortia, strategic plans, and collaborative research programs have been established that either directly or indirectly support the use and improvement of CHIVIM for influenza vaccine development, for example, Inno4Vac, the CIDRAP (University of Minnesota Center for Infectious Disease Research and Policy) universal influenza vaccine roadmap, the NIAID Universal Influenza Strategic Plan, and the NIAID Collaborative Influenza Vaccine Innovation Centers (CIVICs).

Importantly, the efforts to set up a SARS‐CoV‐2 human challenge model resulted in discourse and publication of scientific, regulatory, and ethical considerations and guidelines that also inform the CHIVIM field.

## 3 Session 2: Use Cases for CHIVIM and Regulatory and Ethical Considerations

Industry representatives discussed the utility of CHIVIM in evaluating vaccines in their respective pipelines. The panel discussed various vaccine candidates undergoing clinical trials including quadrivalent influenza vaccine candidates targeting the hemagglutinin (HA) antigen and others exploring H3 and neuraminidase (NA) antigens. Panelists noted a strong preference for benchmarking these vaccines against licensed vaccines rather than relying on CHIVIMs. However, panelists highlighted the potential value of CHIVIMs as a supplementary tool, particularly during the early stages of the vaccine development process (e.g., in down‐selecting mucosal vaccine candidates that are administered differently than current licensed vaccines [4]). Panelists acknowledged several challenges in the use of CHIVIMs, including (1) difficulty of accurately reflecting real‐world vaccine efficacy, (2) need for more standardized and well‐characterized models, (3) cost and time to develop influenza virus for CHIVIM studies, and (4) careful selection of appropriate virus strains and endpoints based on the needs of the study.

Jerry Weir, PhD, from the US Food and Drug Administration, reviewed regulatory considerations for CHIM studies. It was acknowledged that CHIM studies could provide invaluable data that inform the development of next‐generation influenza vaccines, particularly for innovative vaccine candidates that aim to offer broader protection or target emerging influenza strains. CHIM studies should (1) endeavor to accurately reflect the natural exposure route and infectious dose of the virus and (2) determine the correlation between vaccine protection in the challenge model and real‐world efficacy. Researchers should understand how data from the challenge model will be utilized and for which target populations. Discussions with regulatory agencies are necessary to align expectations for how CHIM studies can support vaccine efficacy claims and approval.

Josh Morrison from the nonprofit 1Day Sooner discussed CHIM from the perspective of study volunteers. 1Day Sooner is a grassroots organization whose primary focus is to facilitate the participation of volunteers in high‐impact medical research, particularly COVID‐19 challenge studies, and as such, it actively engages with researchers to contribute to the study design process and facilitates public health advocacy to promote vaccine equity and pandemic preparedness. Participants in medical research have expressed the following expectations and desires regarding their involvement in CHIM studies: (1) increased compensation, (2) enhanced comfort during the study period, (3) greater convenience and less disruption to their daily lives, and (4) a broad social benefit arising from their participation in medical research.

Claudia Emerson, PhD, from McMaster University, presented some ethical considerations for CHIM studies. Although ethics guidance for CHIM studies has a rich history, the World Health Organization (WHO) released key criteria for the ethical acceptability of COVID‐19 CHIMs in 2021 [5], followed by guidance on the ethical conduct of CHIM studies in 2022 [6]. Key criteria outlined for the ethical acceptability of COVID‐19 CHIMs include (1) scientific justification; (2) assessment of risks and potential benefits; (3) consultation and engagement with the public as well as relevant experts and policymakers; (4) close coordination between researchers, funders, policymakers, and regulators; (5) selection of sites where the research can be conducted to the highest scientific, clinical, and ethical standards; (6) selection of study participants that limits and minimizes risk; (7) expert review by a specialized independent committee; and (8) a rigorous informed consent process. A recent SARS‐CoV‐2 human challenge study demonstrated the value of collaboration between academia, industry, and government including advanced coordination, a risk mitigation strategy, public engagement, and strong ethical oversight and governance, which helped inspire confidence in the process and outcomes of CHIMs.

## 4 Session 3: Innovation of CHIVIM

Scientific innovations in CHIVIM studies include the study of viral aerosols and the potential use of CHIVIM for studying viral transmission. To ascertain whether COVID‐19 infection and other phenotypical differences (e.g., infection risk) may alter airborne droplet generation from the airways during breathing, Chad Roy, PhD, from Tulane University, presented data from two studies in human and nonhuman primates (NHPs) [7]. Data from NHP experimental SARS‐CoV‐2 infections showed a rapid increase of the number of exhaled breath particles that correlated with increasing nasal viral concentrations and a return to preexposure numbers during convalescence and viral subsidence. Exhaled breath particles decreased to a more respirable size as numbers increased and viral loading reached its zenith in the animal infections. Particle size distributions returned to normal size once the animals convalesced, suggesting that changes in particle size can impact the distance the particles travel and their potential to infect naïve individuals [7]. Anika Singanayagam, PhD, from Imperial College London, presented viral emissions data from a first‐in‐human SARS‐CoV‐2 experimental infection study in which unvaccinated, seronegative healthy adults aged 18–30 years were intranasally inoculated with a pre‐Alpha (B.1 with D614G) SARS‐CoV‐2 virus [8]. Data from this study revealed that those who became infected emitted large quantities of virus into the air, breath, and contaminated surfaces; however, the extent of viral emissions was heterogeneous between individuals. Symptom scores did not strongly correlate with emissions [7]. In contrast, viral concentration in the nose showed a more substantial correlation with emissions than viral load in the throat [7]. When comparing these findings to natural community‐acquired SARS‐CoV‐2 infection, the majority of infectious virus shedding began with the onset of mild symptoms in both the CHIM and community settings, and the quantity of airborne virus collected in real‐world studies was comparable to that found in the CHIM study. To test the hypothesis that aerosols are the dominant mode of transmission for influenza, Don Milton, MD, DrPH, from the University of Maryland, College Park, and colleagues performed a controlled human influenza virus infection transmission trial (CHIVITT) as a component of their larger evaluating modes of influenza transmission (EMIT) study. Unexpectedly, no viral RNA was detected through in‐room bioaerosol sampling, and a near absence of onward transmission was observed from CHIM‐infected individuals to naïve recipients [9]. An ongoing NIH‐funded study (EMIT‐2) at the University of Maryland seeks to unravel the complexities of influenza transmission and validate or challenge the central hypothesis of aerosol dominance in viral spread using community‐acquired “natural” influenza virus infected individuals as donors. Donors and recipients participate in social activities for up to 60 h in a ~90 m^3^ room with 0.2–0.5 air changes per hour and controlled temperature and relative humidity. Concordant results were obtained in a pilot study conducted by the Center for Transmission of Airborne Pathogens (CTAP) at Emory University. As presented by Dr. Anice Lowen, participants in a controlled environment with limited air volume and minimal air exchange were subject to H3N2 influenza virus exposures by engaging in social activities in the presence of a nasally inoculated, infected donor. Social activities (e.g., playing games) were performed over multiple days, and as of November 2023, none of the exposed recipients had acquired an influenza infection, suggesting a need to optimize the study design for better modeling of transmission (unpublished data). Considerations for optimization include use of different GMP manufacturing processes for influenza challenge strains (e.g., non‐egg based), as well as carefully controlled aerosol delivery to donors. During the panel discussion, the comparison was drawn between viral shedding and transmission in natural community‐acquired cases and that observed in CHIVIMs. Notable differences have been described in the patterns and quantities of viral shedding for influenza, suggesting that further research is needed to fully understand these discrepancies. The panelists also agreed that more research is needed to clearly pinpoint the conditions and enrollment criteria required to enable experimental transmission.

Presentations describing advances in mucosal sampling and immunological assays and how they have the potential to innovate in CHIM studies were given. Felicity Liew, PhD, from Imperial College London, presented data that identified elevated activation of neutrophils at baseline as a driving factor for susceptibility to respiratory syncytial virus (RSV) infection in a controlled human RSV infection model [10]. These findings suggest that baseline mucosal conditions or the immunological “tone” of the mucosa, rather than systemic immune factors, may be a better predictor of infection outcomes after RSV exposure. In a different clinical study, young healthy adults received a quadrivalent live‐attenuated influenza vaccine (LAIV), and detailed analyses of immune activation after vaccination revealed that mucosal IgA and serum IgG were differentially induced among individuals, who diverged into “serum responders” and “nasal responders.” Mucosal IgA responses were associated with stronger early innate responses in the upper respiratory tract and less viral shedding compared to plasma responses, indicating that mucosal correlates of protection need to be considered when developing vaccines for influenza [11]. Simon Jochems, PhD, from Leiden University Medical Center, presented an extensive analysis of nasal immune cells from COVID‐19 patients and controls using cytometry by time of flight (CyTOF), which highlighted sustained CD8^+^ tissue‐resident memory (TRM) T‐cell activation in infected individuals [12]. SARS‐CoV‐2‐specific immunodominant T‐cell clones comprising more than 10% of reads were found in the nasal mucosa, indicating a very targeted immune response [12]. The findings from this study suggest that nasal TRM T cells could be a relevant correlate of protection for influenza and other respiratory viruses. Samuel Kazer, PhD, from Boston Children's Hospital and Harvard Medical School, presented scRNA‐seq data on nasopharyngeal swabs collected from healthy and SARS‐CoV‐2‐infected participants [13]. Whereas nasal epithelial cells obtained from mild and moderate COVID‐19 cases expressed antiviral/interferon‐responsive genes, epithelial cells in severe COVID‐19 cases showed a significantly diminished antiviral response despite having equivalent viral loads [13], suggesting that a deficiency of nasal epithelial antiviral immunity may predispose an individual to severe COVID‐19. Altogether, these presentations highlighted the recent immunological advancements that can be applied to CHIVIM studies, which should lead to a deeper understanding of how the nasal mucosal immune system responds to influenza viruses, both at the cellular and tissue scales. However, additional work is required to standardize effective measurements of immune responses at mucosal surfaces, and more research on mucosal immunity in general is recommended. Lastly, a significant concern was raised as to how findings from controlled human infection studies in healthy young adults apply to other age groups such as older adults or children and to individuals with different health statuses. Researchers should also consider using immunological markers to identify individuals more susceptible to infection, as well as the ethical implications of selecting specific subgroups for CHIVIMs.

## 5 Session 4: CHIVIM Networks and Standardization

A key recommendation from the 2018 London meeting was to work towards increasing capacity for CHIVIM studies. Important progress has been made in this area. A successful multicenter CHIVIM study funded by NIAID was completed in 2019, in which four clinical sites evaluated the association of baseline hemagglutination inhibition (HAI) and neutralizing antibody titers on symptomatic infection with the H1N1pdm09 challenge virus developed by the Laboratory of Infectious Diseases (LID) at NIAID [14]. More recently, NIAID has developed a reverse genetics‐derived clade 3c3a A/Texas/71/2017 H3N2 challenge virus and has completed a dose‐finding study under the CIVICs program (clinicaltrials.gov identifier NCT04978454). This virus was found to have an attack rate of 78% when administered at a dose of 10^6^ TCID_50_. The A/Texas/71/2017 H3N2 challenge virus and associated reagents are now available to the global scientific community [15], and an A/Arkansas/08/2020 (clade 6B.1A.5a.2) pH1N1 challenge virus has been manufactured and will be evaluated in a dose‐finding clinical trial in 2024. The LID at NIAID is also developing a library of GMP‐manufactured influenza challenge viruses that includes contemporary and historical seasonal influenza A viruses, influenza B, and several low‐pathogenicity avian influenza (LPAI) viruses. Even with these accomplishments, it was proposed that a more coordinated, adequately funded consortium is needed in the United States for increasing capacity to conduct CHIVIM trials at all stages of clinical development. Outside the United States, significant efforts towards increased capacity have been made. In the European Union, the CHIMICHURRI group within the Inno4Vac vaccine development consortium [16] manufacture and clinical evaluation of challenge agents. Establishment of novel CHIMs to enable investigation of immunity in the lower respiratory tract and in specific populations, for example, older adults, is planned, as well as addressing regulatory hurdles to more general acceptance of CHIMs in development of vaccines, therapeutics, and other interventions. The Doherty Institute in Melbourne is planning the first CHIVIM study to be conducted in Australia to be performed with the A/Texas H3N2 challenge strain developed by NIAID. The challenge of specific regulatory hurdles in certain countries was highlighted, for example, the need for lengthy reviews for import permits and special review needed for use of a reverse genetics‐derived challenge agent.

The discussions of standardization of CHIVIM covered strain selection, manufacturing, reagent access, clinical protocols, and immunological assays. It was agreed that a diverse range of challenge strains should be developed to allow evaluation of a range of vaccine and therapeutics approaches, in different populations, with different levels of preexisting immunity. Sharing of strains is important for validation of the CHIVIM conducted at multiple centers. It was also agreed that challenge strains do not necessarily need to be produced using the exact same process but should be conducted according to GMP and could be accelerated by simplifying the manufacturing process in some cases. To improve access and capacity for CHIVIM, reagents to facilitate manufacture, for example, cell lines and plasmids, should be made widely available. This is an important aspect of the influenza challenge material sharing process recently established by NIAID. Access to clinical specimens/biobanks from previous CHIVIM studies should also be improved to facilitate future studies.

Considerations for standardization of clinical protocols for CHIVIM include route and method of delivery of the challenge virus, whether or not to prescreen volunteers for HAI titers or other immunologic parameters, dose escalation practices, and sampling methods. There was agreement to come to consensus on disease definitions and recommendations to have a meeting of experts to look at data and publish recommendations for standards. Although clinical study designs are specific to the particular research question, intervention, and population being evaluated, it was agreed that a standardized sampling schedule would be beneficial across CHIVIM studies for purposes of comparison, with the caveat that associated costs and safety of sampling methods need to be carefully considered. The feasibility and acceptability of lower respiratory tract sampling was discussed. Bronchoalveolar lavage (BAL) could be considered, but not as a routine procedure. Caution with this method of sampling is warranted, as it may have an impact on study readouts. For example, in the past, collection of BAL resulted in febrile illness in some study participants. Possible impacts on immunological assessments should be considered with intensive repeated sampling of the nasal mucosa. Some investigators recommended a standard sequence of sample collection to address this. It is also known that the circadian rhythms affect immune responses, so time of day of collection of respiratory tract specimens needs to be considered.

Standardization of immunological assays where possible is important to allow comparison of different candidate vaccines and therapeutics and also for establishment of correlates of protection. The FLUCOP consortium for the standardization and development of assays for assessment of influenza vaccine correlates of protection [17] has made significant progress in this area. Harmonized protocols for serological assays including HAI and virus microneutralization [18] and neuraminidase inhibition enzyme–linked lectin assay (NAI ELLA) [19], as well as for cell‐mediated immunity assays including IFNγ ELISPOT [20], and intracellular cytokine staining (ICS) for CD4^+^ T‐cell responses [21] have been developed, and protocols are being published as they are finalized. Shared critical reagents and standards are available. The involvement of pharmaceutical industry partners in the consortium enables conduct of clinical studies to generate specimens that can be used for assay development. Other consortia that are supporting efforts for assay standardization include Inno4Vac and CONSISE (Consortium for the Standardization of Influenza Sero‐epidemiology). The importance of development of standardized assays with accessible standards and reagents for analysis of mucosal immune responses for CHIVIM studies and influenza vaccine development was emphasized. This field can benefit from and contribute to efforts being applied in this regard to the development of next generation SARS‐CoV‐2 vaccines [22].

## 6 Session 5: Defining Success Using CHIVIM in Vaccine Development

The themes from the preceding discussions at the meeting were framed in the question of how to define success for the use of CHIVIM as a tool in vaccine development. Important lessons can be learned from past CHIVIM studies conducted for the development of antivirals and earlier vaccine studies conducted in the 1970s. The CHIVIM experience from the evaluation of neuraminidase inhibitors—where incidence of febrile illness, upper respiratory symptoms, and also cough, were better than that reported in more recent CHIVIM studies—was used to illustrate the point that the models need to be more closely replicate community‐acquired influenza illness for which patients will seek medical care. It was proposed that in order to achieve this, the inhalation route of inoculation should be explored. It was argued that CHIVIM may not be appropriate for studying influenza virus transmission, which can be performed in real‐world settings, with naturally infected individuals.

The importance of “the fit for purpose” approach for using CHIVIM in vaccine development was emphasized. There are different considerations for the efficient conduct of late‐stage clinical trials to support vaccine licensure than for natural history studies that aim to study immunity and correlates of protection in detail. The use case of the studies should inform objectives and endpoints.

Rapid generation of challenge strains and the agility to quickly pivot to making new strains available would allow the use of CHIVIM trials to provide data that can supplement phase 3 field trials in the event of low levels of circulation of certain strains of interest during the conduct of efficacy trials. From a regulatory perspective, CHIVIMs provide an interesting way to supplement field trial data, and provide valuable data for the evaluation of vaccines based on antigens other than HA, that cannot rely on HAI or neutralizing antibody readouts. CHIVIMs can play an important role in proof of concepts and in de‐risking vaccine development. Although CHIVIM studies are not on the path to licensure for new vaccines, it was acknowledged that work in this area is still very important for vaccine development and that such studies can provide useful supporting data that can complement data obtained from phase 1 clinical trials: They can aid in the determination of which assays will be useful to take forward for late‐stage clinical trials, they can provide important data for novel “next‐generation” vaccine approaches, including mucosal vaccines and evaluation of novel adjuvants, and they can play and important role in the establishment of correlates of protection.

For the development of more broadly protective, more “universal” influenza vaccines, it will be critical for scientists and regulators to come to a consensus on the definition of breadth and to determine the types of data that will be supportive for claims of broad protection. CHIVIMs could provide evidence of broader protection for vaccines based on highly conserved targets using a diverse panel of viruses. It was suggested that in addition to efficacy against seasonal influenza, data from a set of models, including human challenge with LPAI strains and historical strains, as well as a diverse selection of more contemporary strains, along with supportive data from animal studies could be used to claim broad protection. The usefulness of head‐to‐head comparisons of different candidate vaccines in CHIVIM trials was proposed, and it was suggested that this may be possible with expanded capacity and the ability to reproducibly conduct multicenter CHIVIM studies.

It was acknowledged that there are several areas in the CHIVIM field that are not being adequately addressed, including conducting CHIVIM studies in demographically diverse cohorts, studies to further define determinants of susceptibility to influenza infection and disease, and aerosol methods of delivery of challenge virus to more accurately recapitulate natural infection and induce more severe illness in volunteers (see Table 1 for key meeting findings and future directions).

**TABLE 1:** Key takeaways and future directions.

Key takeaways from the meeting
CHIVIM are not a regulatory requirement for licensure of new influenza vaccines and cannot replace field efficacy trials, but they can provide useful supportive data
Hesitancy by vaccine developers to use CHIVIM for de‐risking clinical development
Use case of CHIVIM will determine study objectives and endpoints
Current models do not accurately recapitulate natural infection and disease
CHIVIM are useful for study of natural history of influenza infection and study of immunity
Significant progress has been made in expanding capacity to conduct CHIVIM studies
Manufacturing of challenge viruses can be streamlined
Sharing of challenge strains, assays, reagents, and standards is critical to support advances in the field
There is much interest in the area of aerosol/inhalation modes of delivery of virus
Future directions
Continue to develop infrastructure to support human challenge studies with robust funding for clinical resources
Develop consensus on disease definitions
Clear guidance from regulators on the role and expectations for data from human challenge studies in vaccine development and path to licensure
Exploration of different disease endpoints
Careful evaluation of targeted delivery of challenge virus to lower parts of the respiratory tract
Continued efforts to expand capacity and evaluate CHIVIM in demographically diverse populations
Careful evaluation of performing CHIVIM trials in special populations
Development of consensus of definition of broad protection and how to generate data to support this claim
Continued efforts to harmonize assays for evaluation of candidate vaccines
Continue to improve engagement with CHIVIM trial volunteers
Focus on mucosal vaccines and assays to evaluate them
Work towards establishment of correlates of protection or novel vaccine approaches
Continued collaboration between stakeholders to improve CHIVIM and advance the development of improved vaccines and other medical countermeasures

## Author Contributions

**M. Chelsea Lane:** conceptualization, writing–original draft, writing–review and editing, project administration, supervision. **Catherine J. Luke:** conceptualization, writing–original draft, writing–review and editing, project administration, supervision. **Joseph Bresee:** conceptualization, writing–review and editing. **Vivien G. Dugan:** conceptualization, writing–review and editing. **Diane J. Post:** conceptualization, writing–review and editing, resources. **Julie Schafer:** conceptualization, writing–review and editing. **Paul C. Roberts:** conceptualization, writing–review and editing, supervision. **David E. Wentworth:** conceptualization, writing–review and editing. **Michael G. Ison:** conceptualization, writing–original draft, writing–review and editing, project administration, supervision, resources.

## Conflicts of Interest

The authors declare no conflicts of interest.

### Peer Review

The peer review history for this article is available at https://www.webofscience.com/api/gateway/wos/peer‐review/10.1111/irv.13358.

## Appendix A CHIVIM Studies: Current Status and Future Directions for Innovation

**November 13–14, 2023**

**Meeting Objectives**

- Review the progress of CHIVIM studies since the 2018 meeting in London
- Discuss the use cases for CHIVIM studies, as well as the regulatory and ethical considerations
- Present updates for innovation of CHIVIM, and discuss current limitations
- Present updates from CHIVIM networks and efforts for model standardization
- Provide historical perspectives from prior CHIVIM studies, and discuss parameters for defining success using CHIVIM in vaccine development

**Agenda:**

**Table:**

Monday, November 13th		
Time	Session/topic	Speaker/moderator
8:00–8:30	Breakfast & Registration (Lobby)	
**Session 1: Welcome, highlights, and opening keynote**		
8:30–8:45	Welcome and opening address	Emily Erbelding Mike Ison
8:45–9:30	Opening keynote	Christopher Chiu
**Session 2: Use cases for CHIVIM and regulatory and ethical considerations** **Session 2 co‐moderators: Julie Schafer, Joe Bresee** Product developer and research needs for evaluating vaccines and therapeutics Use of CHIVIM for evaluating pandemic and universal vaccines Selection of appropriate participants and CHIVIM in broader risk groups (e.g., older adults) Regulatory and ethical considerations Specific issues associated with use of data in low‐ and middle‐income countries		
9:30–10:30	Panel discussion 1. Industry perspectives	Sean Tucker Raffael Nachbagauer Vivek Shinde Pamuk Bilsel
10:30–11:00	**Coffee break and group photo**	
11:00–11:35	Short talks: Regulatory and ethical considerations	Jerry Weir Josh Morrison Claudia Emerson
11:35–12:00	Panel discussion 2. Regulatory and ethical considerations	Jerry Weir Marco Cavaleri Josh Morrison Claudia Emerson
12:00–13:00	**Lunch break**	
**Session 3: Innovation of CHIVIM** **Session 3 co‐moderators: Chelsea Lane, Diane Post** New delivery approaches Use of CHIVIM for assessing transmission and transmission blocking New technologies (e.g., viral detection from breath and air) Mucosal sampling and assays Innate immune screening and susceptibility to infection		
13:00–13:15	Exposure route effects outcome in viral disease: Case study of COVID‐19 animal model development	Chad Roy
13:15–13:30	Modeling influenza virus transmission using controlled infection: Challenges and opportunities	Anice Lowen
13:30–13:45	Evaluating modes of influenza infection: RCT design, results, implications, and ongoing trial	Don Milton
13:45–14:00	Collecting viral emissions from the air and environment after human challenge	Anika Singanayagam
14:00–14:15	Compartmentalization of the mucosal antibody response to respiratory infection: *A call‐to‐arms for mucosal antibodies*	Felicity Liew
14:15–14:30	Detecting antigen‐specific T cells in the upper respiratory tract using minimally‐invasive sampling	Simon Jochems
14:30–14:45	Systems‐level view of the nasal mucosa during viral infection	Samuel Kazer
14:45–15:10	**Coffee break**	
15:10–16:10	Panel discussion and audience comments/questions	Session #3 presenters
16:10–16:30	Day 1 takeaway: Conferences IO feedback from all participants	Diane Post, Chelsea Lane
16:30–16:45	Recap of Day 1	Vivien Dugan
17:00	**Adjourn (Day 1)**	

Tuesday, November 14th		
Time	Session/topic	Speaker/moderator
8:00–8:30	Breakfast (lobby)	
8:30–8:40	Plan for Day 2	Mike Ison
**Session 4: CHIVIM networks and standardization** **Session 4 co‐moderators: Catherine Luke, Chris Roberts, Dave Wentworth** Data presentations from CHIVIM networks and their capabilities Standardization of strain selection and manufacturing (and CHIVIM reagent access) Standardization of CHIVIM clinical protocols (e.g., routes of challenge administration and delivery methods, definition of disease) Standardization of traditional serologic and cellular adaptive immune response assays		
8:40–9:05	NIAID collaborative influenza vaccine innovation centers and infectious diseases clinical research consortium studies	Kathy Neuzil
9:05–9:20	Human influenza challenge models: Meeting the demands of the future	Matt Memoli
9:20–9:40	CHIVIM plans at the Doherty Institute in Australia	Kanta Subbarao
9:40–10:40	Panel discussion 1. Standardization of strain selection and manufacturing and CHIVIM reagent access	Dave Wentworth Matt Memoli Richard Webby Derek Smith Florian Krammer Dan Stoughton Andrew Catchpole
10:40–11:00	**Coffee break**	
11:00–12:00	Panel discussion 2. Standardization of CHIVIM clinical trial protocols	Kathy Neuzil Christopher Chiu Dan Hoft Chris Woods Matt Memoli
12:00–13:00	**Lunch**	
13:00–13:40	Panel discussion 3. Standardization of immunological assays	Kanta Subbarao Othmar Engelhardt Rebecca Cox
**Session 5: Defining success using CHIVIM in vaccine development** **Session 5 co‐moderators: Mike Ison, Marianne Stanford**		
13:40–14:20	Panel discussion	Fred Hayden Hanna Golding Marco Cavaleri Andrew Catchpole Sean Tucker Jeff Taubenberger Justin Ortiz
14:20–14:40	Day 2 takeaway: Conferences IO feedback from all participants	Mike Ison Marianne Stanford
14:40–14:55	Recap of Day 2	Catherine Luke
14:55–15:00	**Closing and thanks**	Catherine Luke Chelsea Lane
